# At-Risk and Recent-Onset Type 1 Diabetic Subjects Have Increased Apoptosis in the CD4+CD25+^high^ T-Cell Fraction

**DOI:** 10.1371/journal.pone.0000146

**Published:** 2007-01-03

**Authors:** Sanja Glisic-Milosavljevic, Jill Waukau, Parthav Jailwala, Srikanta Jana, Huoy-Jii Khoo, Hope Albertz, Jeffrey Woodliff, Marilyn Koppen, Ramin Alemzadeh, William Hagopian, Soumitra Ghosh

**Affiliations:** 1 The Max McGee National Center for Juvenile Diabetes and Human Molecular Genetics Center, Medical College of Wisconsin and the Children's Research Institute of the Children's Hospital of Wisconsin, Milwaukee, Wisconsin, United States of America; 2 Blood Center of Wisconsin, Medical College of Wisconsin, Milwaukee, Wisconsin, United States of America; 3 Flow Cytometry Core Facility, Department of Pediatrics, Medical College of Wisconsin, Milwaukee, Wisconsin, United States of America; 4 Children's Hospital of Wisconsin Diabetes Center, Pediatric Endocrinology and Metabolism, Medical College of Wisconsin, Milwaukee, Wisconsin, United States of America; 5 Pacific Northwest Research Institute, Seattle, Washington, United States of America; University of Oslo, Norway

## Abstract

**Background:**

In experimental models, Type 1 diabetes T1D can be prevented by adoptive transfer of CD4+CD25+ FoxP3+ suppressor or regulatory T cells. Recent studies have found a suppression defect of CD4+CD25+^high^ T cells in human disease. In this study we measure apoptosis of CD4+CD25+^high^ T cells to see if it could contribute to reduced suppressive activity of these cells.

**Methods and Findings:**

T-cell apoptosis was evaluated in children and adolescent 35 females/40 males subjects comprising recent-onset and long-standing T1D subjects and their first-degree relatives, who are at variable risk to develop T1D. YOPRO1/7AAD and intracellular staining of the active form of caspase 3 were used to evaluate apoptosis. Isolated CD4+CD25+^high^ and CD4+CD25− T cells were co-cultured in a suppression assay to assess the function of the former cells. We found that recent-onset T1D subjects show increased apoptosis of CD4+CD25+^high^ T cells when compared to both control and long-standing T1D subjects p<0.0001 for both groups. Subjects at high risk for developing T1D 2–3Ab+ve show a similar trend p<0.02 and p<0.01, respectively. On the contrary, in long-standing T1D and T2D subjects, CD4+CD25+^high^ T cell apoptosis is at the same level as in control subjects p = NS. Simultaneous intracellular staining of the active form of caspase 3 and FoxP3 confirmed recent-onset FoxP3+ve CD4+CD25+^high^ T cells committed to apoptosis at a higher percentage 15.3±2.2 compared to FoxP3+ve CD4+CD25+^high^ T cells in control subjects 6.1±1.7 p<0.002. Compared to control subjects, both recent-onset T1D and high at-risk subjects had significantly decreased function of CD4+CD25+^high^ T cells p = 0.0007 and p = 0.007, respectively.

**Conclusions:**

There is a higher level of ongoing apoptosis in CD4+CD25+^high^ T cells in recent-onset T1D subjects and in subjects at high risk for the disease. This high level of CD4+CD25+^high^ T-cell apoptosis could be a contributing factor to markedly decreased suppressive potential of these cells in recent-onset T1D subjects.

## Introduction

Type 1 diabetes T1D is an autoimmune disorder in which autoreactive T cells attack pancreatic β-cells, leading to a lifelong dependency on insulin. Twenty years ago, T1D was hypothesized to be a chronic autoimmune disorder by Eisenbarth[1]. Considerable progress has been made in understanding the pathogenesis of autoimmune disorders since then, but not enough to prevent or reverse the disease. The abrogation of central and peripheral tolerance underpins the genesis of all autoimmune diseases[2]. At the heart of these processes lies apoptosis. In central tolerance, autoreactive T cells are deleted in the thymus through apoptosis pathways. Most studies in murine T1D models have found that autoreactive T cells are resistant to apoptosis[3]. This predisposes an individual to autoimmune disease as autoreactive T cells find their way to the periphery and attack the target organ. Only a few studies have investigated apoptosis of T cells in the periphery in T1D patients and nearly all before the era of regulatory T cells[4]–[6]. There is some evidence that resistance to apoptosis of autoreactive T cells contributes to human T1D [6]. In parallel, the thymus produces regulatory T cells whose main action is in the periphery control of development, trafficking and proliferation of responder T cells. Though there are other mechanisms for peripheral tolerance[7], the role of CD4+CD25+^high^ T cells with regulatory function Tregs has recently attracted considerable attention.

Although the existence and function of regulatory T cells is confirmed in both *in vivo* and *in vitro* experiments, exact identification and isolation has been and continues to be a problem. The FACS isolation method that gives the high number of most potent Tregs is based on the highest surface expression of CD25 IL2-Ralpha[8]. These CD4+CD25+^high^ T cells are enriched in cells with regulatory properties. It has been shown in animal models that CD4+CD25+^high^ T cells can prevent murine T1D[9]–[11] bringing in new hope for the prevention of human autoimmune disease. However, CD4+CD25+^high^ also include activated CD4+CD25+ T cells that do not generally show regulatory properties. Nevertheless, high expression of CD25 on CD4+ cells has been shown to mark a small number of highly suppressive T cells. Although a specific molecule for regulatory T cells is lacking, the transcription factor FoxP3 - forkhead box P3, has been identified as necessary for their development[12]. It has also been confirmed that cells with regulatory function constitutively express high levels of the FoxP3, but this is an intracellular transcription factor, so isolation of live human cells based on FoxP3 staining is not possible. Recently however, some papers have shown correlation of high expression of FoxP3 and low or no expression of IL-7R alpha CD127low/- on the cell surface[13], [14] Humans without functional FoxP3 protein have an autoimmune disorder called IPEX with auto-antibodies against a diversity of organs[15]. However, there are still controversial data about FoxP3 involvement in regulatory function[16].

Several studies with healthy control subjects and T1D patients have reported decreased regulatory function of CD4+CD25+^high^ T cells in T1D patients[17]–[20]. Despite accumulating data about the dysfunction of CD4+CD25+^high^ T cells in T1D patients, there is no compelling evidence identifying an underlying cause. Spontaneous apoptosis of Tregs in human T1D patients as a mechanism has not been fully investigated.

In this study, we measured apoptosis of CD4+CD25+^high^ T cells in relation to their function. Healthy control and several T1D-related clinical groups were studied. These included recent-onset T1D subjects and unrelated, unaffected subjects with one, two and three positive T1D specific auto-antibodies aAbs to GAD, IA-2 and insulin. Those with two or more aAbs are at higher risk for developing T1D in the future. We also studied longstanding T1D and T2D patients. Our results implicate a role of increased apoptosis of CD4+CD25+^high^ T cells in the pathogenesis of human T1D. Furthermore, intriguing data presented here shows that Tregs FoxP3+ve T cells within this population contribute most to the increased apoptosis. However, a strong inverse relationship between apoptosis of CD4+CD25+^high^ T cells and their function needs to be investigated on a larger sample size in different clinical groups.

## Methods

Seventy-five Wisconsin study subjects 67 Caucasian, 5 Asian, 2 African-American and 1 Hispanic of both genders 35 females and 40 males were recruited through the diabetes clinics at Children's Hospital of Wisconsin CHW, Froedtert Hospital FMLH and other local hospitals and clinics that had over 1600 T1D families, through ascertainment of a proband with the T1D onset before age of 17. In families with a child proband, siblings and parents were also asked to join the study. However, just one person from each family was included in our study. Control subjects were recruited by posting flyers in areas of the hospitals that might be visited by potential “healthy” subjects, i.e. orthopedic, dental, and eye clinics. Inclusion criteria for control subjects were random blood glucose less then 110 mg/dl, no history of any autoimmune disorder including T1D in the family and an absence of T1D- specific aAbs. Although healthy subjects of all ages were included n = 28, age-matched control subjects formed a separate clinical group n = 10 because of the possible age effect on number and function of isolated CD4+CD25+high T cells[21]. At-risk subjects n = 17 were first-degree relatives of T1D patients, but unrelated to the specific probands included in this study. T1D subjects were defined according to accepted criteria: high blood glucose levels ≥200 mg/dl with symptoms of diabetes confirmed by a physician, according to WHO criteria[22]. Recent-onset T1D subjects n = 14 were recruited after metabolic stabilization and within one year of diagnosis. Long-standing T1D subjects n = 12 were diagnosed at least two years earlier. Random glucose levels at the time of recruitment in this group of subjects were at comparable levels to the glucose levels measured in recent-onset T1D subjects Table 1. Long-standing T2D subjects n = 4 were also diagnosed at least two years earlier. They were included in the study as a group with non-autoimmune diabetes. Subjects or their parents guardians provided written informed consent and completed a questionnaire.

**Table 1:**
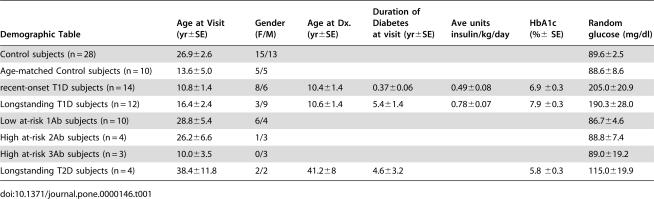
Demographic data of subjects involved in the study

Demographic Table	Age at Visit yr±SE	Gender F/M	Age at Dx. yr±SE	Duration of Diabetes at visit yr±SE	Ave units insulin/kg/day	HbA1c %± SE	Random glucose mg/dl
Control subjects n = 28	26.9±2.6	15/13					89.6±2.5
Age-matched Control subjects n = 10	13.6±5.0	5/5					88.6±8.6
recent-onset T1D subjects n = 14	10.8±1.4	8/6	10.4±1.4	0.37±0.06	0.49±0.08	6.9 ±0.3	205.0±20.9
Longstanding T1D subjects n = 12	16.4±2.4	3/9	10.6±1.4	5.4±1.4	0.78±0.07	7.9 ±0.3	190.3±28.0
Low at-risk 1Ab subjects n = 10	28.8±5.4	6/4					86.7±4.6
High at-risk 2Ab subjects n = 4	26.2±6.6	1/3					88.8±7.4
High at-risk 3Ab subjects n = 3	10.0±3.5	0/3					89.0±19.2
Longstanding T2D subjects n = 4	38.4±11.8	2/2	41.2±8	4.6±3.2		5.8 ±0.3	115.0±19.9

All participants had aAbs and glucose levels measured at the recruitment visit. An accurate method for the detection of multiple T1D auto-antibodies developed by Hagopian and collaborators was employed[23]. His laboratory participated in the 2005 CDC/IDS Diabetes Autoantibody Standardization Program, with sensitivity/specificity of 94%/94%, 80%/93% and 76%/93% for GAD Ab, IA2 Ab and IAA, respectively.

Recent-onset and long-standing T1D and T2D subjects had HbA1c and insulin dosage recorded at each visit. Peripheral blood mononuclear cells PBMC were collected using vacutainers with ACD solution B of trisodium citrate and isolation was done using Ficoll-Hypaque density gradient centrifugation following the manufacturer's protocol Amersham Pharmacia, Uppsala, Sweden. Cells were counted and stained with a cocktail of fluorochrome-conjugated monoclonal antibodies in PBS APC-αCD4 clone RPA-T4, APC-Cy7-αCD25 clone M-A251, FITC-αCD14 clone M5E2, FITC-αCD32 clone FLI8.26, FITC-αCD116 clone M5D12, PE-Cy7-CD8 clone RPA-T8 all from BD Pharmingen, San Diego, CA and sorted on FACSAria BD Biosciences, San Diego, CA. Cells were gated first for live lymphocyte, eliminating dead cells and debris. Next, two gates were set up to eliminate non-CD4 T cells FITC- and PECy7-conjugated antibodies. CD4+ T cells were further gated as CD4+CD25- and CD4+CD25+^high^ using the Fluorochrome Minus One FMO method, that allows a more precise definition of cells having fluorescence above the background level[24]. The 1% of cells expressing the highest level of CD25 were collected and defined as CD4+CD25+^high^ T cells, known to be enriched for Tregs[25]. These cells showed expected distribution of non-unique surface markers expected for Tregs data not shown.

Apoptosis in all samples was measured using our recently published method [26]. Briefly, cells were stained with YOPRO1 250 nM for 20 minutes in the dark and 7AAD 250 ng was added 10 minutes before acquiring events. Apoptosis was measured as the percentage of apoptotic cells YOPRO1+ve/7AAD-ve amongst live cells total 7AAD-ve cells comprising both YOPRO1+ve and YOPRO1-ve cells. Using a similar computation, apoptosis was also measured by intracellular staining of the active form of caspase 3 aaCas3, in most cases combined with intracellular staining of the transcription factor FoxP3, constitutively expressed in the Treg subfraction of CD4+CD25+^high^ T cells[27]. Caspase 3 is a key executioner in the apoptosis pathway and detection of its active form is usually a sign of ongoing apoptosis. Before we adopted the method of dual staining of FoxP3 and the active form of caspase 3, we confirmed that the results of single staining for both proteins were similar to results generated by their simultaneous staining data not shown.

The only reliable feature of CD4+CD25+^high^ T cells is their capacity to suppress proliferation of responder T cells. This capacity is measured via a proliferation assay, where responder CD4+CD25− and suppressor within the CD4+CD25+^high^ population T cells are stimulated *in vitro* separately and together in co-culture under defined conditions. The proliferation assay was performed using 10,000 responder T cells CD4+CD25− and 10,000 irradiated PBMCs 5000rad along with 1,000 CD4+CD25+^high^ T cells seeded in a 96-well plate. Stimulation was with aCD3-coated beads 1 ug/ml, 3 beads/cell for 5 days under 5% CO_2_ and saturated humidity. Cells were left in culture after pulsing 1 µCi of [H^3^]-thymidine for an additional 16 hours, then harvested and after adding scintillation liquid, read for counts per minute cpm. Suppression % of Tregs was calculated as [cpm of responders in single culture − cpm of cells in co-culture/cpm of responders in single culture]×100.

The Mann-U-Whitney and Tukey-Kramer tests were used to compare results between clinical groups with p value ≤0.05 considered significant. GraphPad software was used for data presentation. We also performed Kruskal-Wallis in addition to a one-way ANOVA.

## Results

We used a FACS machine for cell separation in this study. The sorter allows specific isolation of a cell population from a heterogeneous T-cell mixture, especially if the surface marker is used as a tag. To minimize the number of activated T cells that express high levels of CD25, we only collected 1% of cells with the highest CD25 expression. CD4+CD25+^high^ T cells.

In all clinical groups studied, two T-cell subsets CD4+CD25− and CD4+CD25+^high^ were collected and analyzed *ex vivo* for 1. proliferation/suppression; 2. apoptosis determination using nucleic acid stains YOPRO1/7AAD; 3. intracellular staining for FoxP3 and 4. intracellular staining for aaCas3. The CD4+CD25− T cells are used as responders in a suppression assay and they also served as an internal control for the measurement of apoptosis.

**Figure 1:**
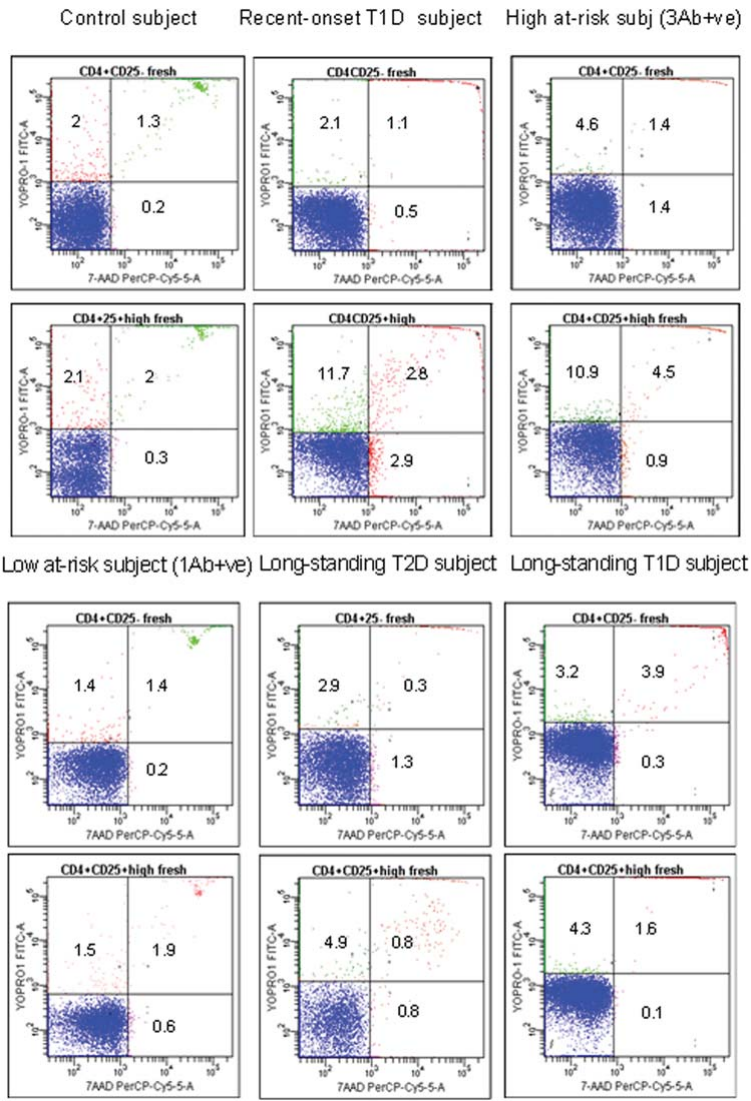
Representative FACS pictures of CD4+CD25− and CD4+CD25+^high^ T cells using YOPRO1/7AAD method from representative subjects of clinical groups. Apoptosis was calculated as the percentage of apoptotic cells YOPRO1+ve/7AAD-ve amongst live cells total 7AAD-ve cells comprising both YOPRO1+ve and YOPRO1-ve cells. This is simply designated as YOPRO1+ve.

**Figure 2:**
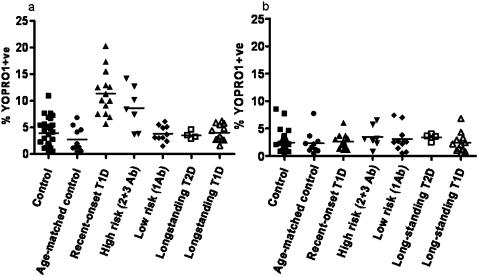
a Apoptosis of CD4+CD25+^high^ T cells in clinical groups measured by YOPRO/7AAD stain combination. CD4+CD25+^high^ T cells in recent-onset and in high at-risk subjects for developing T1D show significantly higher apoptosis levels: Kruskal-Wallis p<0.0001; ANOVA F = 16.07 df 6, 78 p<0.0001. Specifically, Control vs. recent-onset T1D p<0.0001; age-matched control vs. recent-onset p<0.0001; recent-onset T1D vs. long-standing T1D p<0.0001; recent-onset T1D vs. low at-risk p<0.0001; control vs. high at-risk p<0.008; high at-risk vs. long-standing T1D p = 0.01. b Apoptosis of CD4+CD25− T cells measured by YOPRO/7AAD stain combination across studied clinical groups Kruskal-Wallis p = 0.25; ANOVA F = 0.59, df 6, 78, NS. Apoptotic cells are presented on y-axis as YOPRO1+ve.

Within a uniformly short time after FACS isolation, in all studied subjects, fresh CD4+CD25− and CD4+CD25+^high^ T cells were analyzed for apoptosis using the YOPRO1/7AAD stain combination[26]. Representative FACS pictures for each group of patients are presented in Figure 1. CD4+CD25+^high^ T-cell apoptosis was significantly different across the clinical groups Kruskal-Wallis p<0.0001; ANOVA F = 16.07 df 6, 78, p<0.0001, Figure 2a. Based on the Tukey-Kramer post-hoc test, CD4+CD25+^high^ T-cell apoptosis in recent-onset T1D subjects differed significantly from all other clinical groups except from the high at-risk group Figure 2a. Concurrently, CD4+CD25− T cells, which were isolated from all subjects at the same visit, showed no differences in apoptosis levels across the clinical groups Kruskal-Wallis p = 0.25 NS; ANOVA F = 0.73 df 6, 78, p = 0.26; NS; Figure 2b. Apoptosis of CD4+CD25+^high^ is the highest in recent-onset T1D subjects 11.4±1.1 followed by high at-risk subjects 8.6±1.5. The lowest measured CD4+CD25+^high^ T-cell apoptosis level is among the longstanding T2D 3.5±0.4 and is similar to CD4+CD25+^high^ T-cell apoptosis among both the control and longstanding T1D subjects 3.9±0.5 and 4.0±0.5 respectively. When compared to control subjects, CD4+CD25+^high^ T-cell apoptosis of recent-onset T1D showed the strongest statistical difference p<0.0001. We were also able to recruit ten age-matched control subjects to rule out the possibility that increased age was associated with reduced apoptosis of CD4+CD25+^high^ T cells. Compared to the age-matched control group, recent-onset T1D subjects showed a significant increase in CD4+CD25+^high^ T cell apoptosis p<0.0001. While not as noticeable, the high at-risk clinical group both 2 Ab+ and 3 Ab+ also showed a significant increase in CD4+CD25+^high^ T-cell apoptosis when compared to random control subjects p = 0.008.

**Figure 3:**
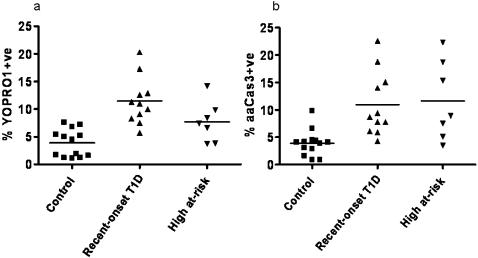
Comparison of apoptosis measurement in the same subjects using two different methods. a CD4+CD25+^high^ T cells apoptosis in three clinical groups using YOPRO/7AAD stain combination; control subjects CD4+CD25+^high^ T cells apoptosis significantly differs from both recent-onset T1D and high at-risk subjects p = 0.0001 and p = 0.01, respectively. Apoptotic cells are presented on y-axis as YOPRO1+ve. b CD4+CD25+^high^ T cells apoptosis in the same subjects using intracellular staining of aaCas3. CD4+CD25+^high^ T cells apoptosis from control subjects significantly differs from both recent-onset T1D and high at-risk subjects p = 0.0006 and p = 0.009, respectively. Spearman's rank correlation coefficient between two apoptosis measurement methods in control and recent-onset T1D subjects is significant r = 0.59 and p = 0.0006. Apoptotic cells are presented on y-axis as aaCas3+ve.

In order to confirm our results using YOPRO1/7AAD, we used a second apoptosis detection method. Thus, an aliquot of isolated T cells was stained for the presence of active caspase 3. Figure 3a and 3b present CD4+CD25+^high^ T-cell apoptosis isolated from the same subjects measured by two different apoptosis assays. The apoptosis results were similar. Furthermore, correlation of apoptosis results measured by these two methods was significant Spearman's rank correlation coefficient r = 0.59 95% CI = 0.28–0.79, p = 0.0006. A higher percentage of CD4+CD25+^high^ T cells in recent-onset T1D subjects produced more active caspase 3 than the same T-cell subtype in control subjects, confirming the ongoing apoptosis detected by YOPRO1/7AAD. Control subjects had significantly lower apoptosis level from both recent-onset T1D and high at-risk subjects as measured by intracellular staining of aaCas3 p = 0.0006 and p = 0.009 respectively.

**Figure 4:**
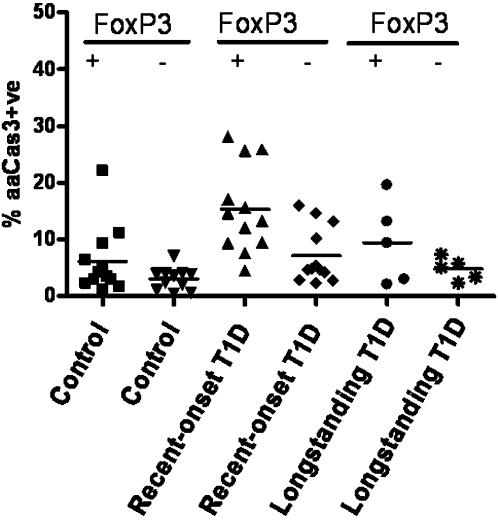
CD4+CD25+^high^ T cells apoptosis levels within FoxP3+ve and FoxP3-ve cell subpopulation across three clinical groups. FoxP3+ve CD4+CD25+^high^ T cells show significantly higher apoptosis level intracellular staining of aaCas3 compared to FoxP3-ve CD4+CD25+^high^ T cells in recent-onset T1D only Kruskal-Wallis, p = 0.0066; recent-onset T1D CD4+CD25+^high^ T cells FoxP3+ve vs. FoxP3-ve p = 0.01; FoxP3+ve CD4+CD25+^high^ T cells control vs. FoxP3+ve CD4+CD25+^high^ T cells recent-onset T1D p<0.002. FoxP3-ve CD4+CD25+^high^ T cells control vs. FoxP3-ve CD4+CD25+^high^ T cells recent-onset T1D p = 0.01. Apoptotic cells are presented on y-axis as aaCas3+ve.

Since CD4+CD25+^high^ T cells comprise FoxP3+ Tregs[27] as well as activated T cells, we checked whether cells that were dying by apoptosis were FoxP3 positive. Simultaneous intracellular staining of the active form of caspase 3 and FoxP3 confirmed recent-onset FoxP3+ve CD4+CD25+^high^ T cells committed to apoptosis at a higher percentage 15.3±2.2 compared to FoxP3+ve CD4+CD25+^high^ T cells in control subjects 6.1±1.7; Figure 4, p<0.002. Of great interest, while FoxP3+ve and FoxP3-ve CD4+CD25+^high^ T cells in both control and longstanding T1D subjects did not show differences in the apoptosis rate, FoxP3+ve CD4+CD25+^high^ in recent-onset T1D subjects were significantly more apoptotic than FoxP3-ve CD4+CD25+^high^ T cells from the same individual Figure 4, p = 0.01.

**Figure 5:**
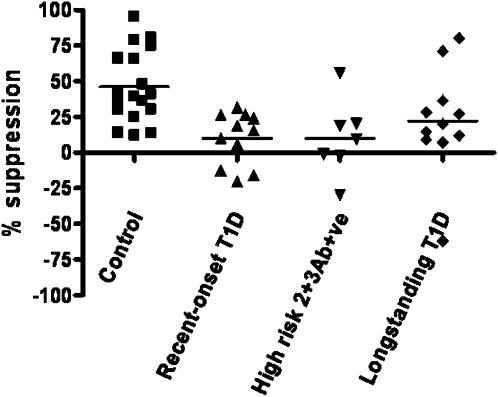
CD4+CD25+^high^ T cells suppressive potential across major clinical groups. Control subjects show statistically higher suppression compared to the other three groups recent-onset T1D, high at-risk and longstanding T1D subjects p = 0.0007, p = 0.007 and p = 0.04, respectively. Standardized suppression assay was performed with 1×10^4^ responder T cells, 10^4^ irradiated PBMCs 5000rad alone and in co-culture with CD4+CD25+^high^ T cells in 1∶10 ratio, stimulated with aCD3 coated beads 1ug/ml, 3 beads/per cell.

FACS-isolated CD4+CD25+^high^ T cells were also tested for their suppressive function using standardized *in vitro* suppression assays. Analysis of CD4+CD25−/CD4+CD25+^high^ T cell co-culture at a ratio of 1∶10 indicated that CD4+CD25+^high^ T cells in control subjects showed average suppression of 46.0.±5.9% while high at-risk and recent-onset T1D subjects showed similar suppression of 10.±9.9% and 9.9±5.5% respectively, Figure 5. Compared to control subjects, both recent-onset T1D and high at-risk subjects had significantly decreased function of CD4+CD25+^high^ T cells p = 0.0007 and p = 0.007, respectively. Although suppressive potential of CD4+CD25+^high^ T cells isolated from longstanding T1D subjects was higher than in recent-onset T1D subjects, it was significantly lower when compared to healthy controls p = 0.04.

One must be aware of other factors associated with increased apoptosis of T cells in the clinical setting. These include ambient glucose[28] and insulin[29] levels. We provide evidence that these factors play only a minor role in our apoptosis results. In our sample, longstanding T1D subjects have similar glucose levels to recent-onset T1D subjects p = 0.52, NS, similar CD4+CD25− T cells apoptosis levels p = 0.21, but completely disparate apoptosis levels of their fresh CD4+CD25+^high^ T cells p<0.0001. Although exogenous insulin levels are significantly different because of the honeymoon phase p = 0.004, our data for CD4+CD25− T cells do not show apoptosis differences, which confirm a non-significant role of insulin or any other factor in the protection from or genesis of apoptosis. Age-dependent differences in the frequency of have been shown before[20]. However, in the present study, no differences in apoptosis of CD4+CD25+^high^ T cells dependent on donor age were observed Mann-Whitney U-test p = 0.19.

## Discussion

In this study, we aimed to measure apoptosis and function of CD4+CD25+^high^ T cells in T1D families and control subjects. Although a recent study shows that 2–3% of the highest CD25-expressing T cells can be safely sorted as Tregs[30], we only collect the top 1%. This is because subjects with ongoing autoimmune disease have more activated T cells that also express CD25, even though these cells possess no regulatory properties. For now, the presence of true Tregs is best defined by their functional activity, which *in vitro*, is monitored by suppression of the proliferation of responder T cells. In fact, recent studies *in vivo* have confirmed that Tregs probably act in a similar manner by inhibiting the interaction between antigen-presenting cells and responder T cells, leading to reduced proliferation of the latter cells[31]. Most groups have reported regulatory T-cell CD4+CD25+^high^ dysfunction in T1D [17], [20]. None have found a cause for this dysfunction.

We present evidence that increased apoptosis of Tregs could potentially play a role in the pathogenesis of human T1D, a major autoimmune disease. Apoptosis was measured in freshly isolated CD4+CD25+^high^ T cells from T1D families and control subjects. The highest levels of apoptosis were found in recent-onset T1D subjects and in individuals at high risk 2+3Ab+ve for disease Figure 2. This important observation was only possible after we developed a novel, sensitive stain combination YOPRO1/7-AAD for the detection of early apoptosis [26]. YOPRO1 is a small molecule that enters the apoptotic cells readily compared to other stains.

Our data suggest that individuals who are positive for one Ab low risk to develop T1D have a low apoptosis level of CD4+CD25+^high^ T cells that is similar to control subjects. The percentage of apoptosis increases with the number of Abs detected in the serum, reaching the highest level in subjects at the onset of the disease staying high during the period immediately following diagnosis. It is during this period that requirements for exogenous insulin drop dramatically and beta cell function improves “honeymoon” phase. Later on, when the autoimmune destruction is complete and insulin requirements increase, CD4+CD25+^high^ T-cell apoptosis goes back to the level detected in healthy control subjects.

Our results with the new stain combination are confirmed with a second, independent, apoptosis detection method on the same samples. The second method was the intracellular staining for the active form of caspase 3. Generated results parallel the apoptosis data in the CD4+CD25+^high^ T-cell fraction using YOPRO1/7-AAD Figure 3. These results also suggest that apoptosis in recent-onset T1D subjects involves pathways with caspase 3. Furthermore, with the simultaneous staining for the active form of caspase 3 and the transcription factor FoxP3, we are able to differentiate apoptosis based on FoxP3 expression in CD4+CD25+^high^ T cells Figure 4. These results suggest that it is the true Tregs FoxP3+CD4+CD25+^high^ T cells that contribute most to the apoptosis seen in highest CD25-expressing T cells.

Even though under intense investigation during the last several years, it has not been completely determined why CD4+CD25+^high^ T cells have reduced *in vitro* suppressive capacity in T1D. Some of the possible causes include insufficient amount of ambient IL2[32], mutations in FoxP3[33] prevention of CD4+CD25+^high^ T cells development or non-functional TGF-βR on the surface of responder T cells [34]. The only report that we are aware of shows increased apoptosis of human CD4+CD25+^high^ T cells after FACS isolation but under specific conditions Fritzsching et al., 2005 [35]. No reports have yet appeared to link increased apoptosis in the unmanipulated CD4+CD25+^high^ T-cell fraction with respect to autoimmune disease.

How does increased apoptosis of Tregs affect their function? Since *in vitro* suppression is the only functional test, we have developed a robust assay Jana et al, in preparation. High suppression in control subjects using our high co-culture ratio ensures that functionally these are true suppressor cells. Significant suppression differences between control and recent-onset T1D subjects following the same experimental conditions, point to a functional impairment of regulatory cells seen in the recent-onset T1D clinical group Figure 5. This result is in agreement with previous studies done with recent-onset T1D patients[17]. Another comprehensive study also reported T1D patients having deficient function of CD4+CD25+^high^ T cells[20].

The novel stain combination for apoptosis measurement used here for the first time in a study involving diabetic patients correlates well with the number of diabetes-specific auto-antibodies. A future longitudinal study on more subjects will give direct proof that ongoing apoptosis of CD4+CD25+^high^ T cells leads to reduced suppression and the beginning of T1D. Although other tests for the prediction of T1D exist family history and HLA genotyping, we believe that this easy-to-use method that captures early apoptosis of CD4+CD25+^high^ T cells may be of value as a marker for individuals at T1D risk and that it opens up a new area in T1D investigation.

## References

[1] Eisenbarth GS (1986). Type I diabetes mellitus. A chronic autoimmune disease.. N Engl J Med.

[2] Mathis D, Benoist C (2004). Back to central tolerance.. Immunity.

[3] Liston A, Lesage S, Gray DH, O'Reilly LA, Strasser A (2004). Generalized resistance to thymic deletion in the NOD mouse; a polygenic trait characterized by defective induction of Bim.. Immunity.

[4] Giordano C, De Maria R, Stassi G, Todaro M, Richiusa P (1995). Defective expression of the apoptosis-inducing CD95 Fas/APO-1 molecule on T and B cells in IDDM.. Diabetologia.

[5] Giordano C, Stassi G, Todaro M, De Maria R, Richiusa P (1995). Low bcl-2 expression and increased spontaneous apoptosis in T-lymphocytes from newly-diagnosed IDDM patients.. Diabetologia.

[6] Dosch H, Cheung RK, Karges W, Pietropaolo M, Becker DJ (1999). Persistent T cell anergy in human type 1 diabetes.. J Immunol.

[7] Buckner JH, Ziegler SF (2004). Regulating the immune system: the induction of regulatory T cells in the periphery.. Arthritis Res Ther.

[8] Sakaguchi S, Sakaguchi N, Asano M, Itoh M, Toda M (1995). Immunologic self-tolerance maintained by activated T cells expressing IL-2 receptor alpha-chains CD25. Breakdown of a single mechanism of self-tolerance causes various autoimmune diseases.. J Immunol.

[9] Tang Q, Henriksen KJ, Bi M, Finger EB, Szot G (2004). In vitro-expanded antigen-specific regulatory T cells suppress autoimmune diabetes.. J Exp Med.

[10] Tarbell KV, Yamazaki S, Olson K, Toy P, Steinman RM (2004). CD25+ CD4+ T cells, expanded with dendritic cells presenting a single autoantigenic peptide, suppress autoimmune diabetes.. J Exp Med.

[11] Lundsgaard D, Holm TL, Hornum L, Markholst H (2005). In vivo control of diabetogenic T-cells by regulatory CD4+CD25+ T-cells expressing Foxp3.. Diabetes.

[12] Sakaguchi S (2005). Naturally arising Foxp3-expressing CD25+CD4+ regulatory T cells in immunological tolerance to self and non-self.. Nat Immunol.

[13] Liu W, Putnam AL, Xu-Yu Z, Szot GL, Lee MR (2006). CD127 expression inversely correlates with FoxP3 and suppressive function of human CD4+ T reg cells.. J Exp Med.

[14] Seddiki N, Santner-Nanan B, Martinson J, Zaunders J, Sasson S (2006). Expression of interleukin IL-2 and IL-7 receptors discriminates between human regulatory and activated T cells.. J Exp Med.

[15] Wildin RS, Smyk-Pearson S, Filipovich AH (2002). Clinical and molecular features of the immunodysregulation, polyendocrinopathy, enteropathy, X linked IPEX syndrome.. J Med Genet.

[16] Allan SE, Passerini L, Bacchetta R, Crellin N, Dai M (2005). The role of 2 FOXP3 isoforms in the generation of human CD4+ Tregs.. J Clin Invest.

[17] Lindley S, Dayan CM, Bishop A, Roep BO, Peakman M (2005). Defective suppressor function in CD4+CD25+ T-cells from patients with type 1 diabetes.. Diabetes.

[18] Kukreja A, Cost G, Marker J, Zhang C, Sun Z (2002). Multiple immuno-regulatory defects in type-1 diabetes.. J Clin Invest.

[19] Putnam AL, Vendrame F, Dotta F, Gottlieb PA (2005). CD4+CD25high regulatory T cells in human autoimmune diabetes.. J Autoimmun.

[20] Brusko TM, Wasserfall CH, Clare-Salzler MJ, Schatz DA, Atkinson MA (2005). Functional defects and the influence of age on the frequency of CD4+ CD25+ T-cells in type 1 diabetes.. Diabetes.

[21] Gregg R, Smith CM, Clark FJ, Dunnion D, Khan N (2005). The number of human peripheral blood CD4+ CD25high regulatory T cells increases with age.. Clin Exp Immunol.

[22] (1999). Definition, diagnosis and classification of diabetes mellitus and its complications. Report of a WHO Consultation, Part I: Diagnosis and Classification of Diabetes Mellitus, Geneva, World Health Organization.

[23] Woo W, LaGasse JM, Zhou Z, Patel R, Palmer JP (2000). A novel high-throughput method for accurate, rapid, and economical measurement of multiple type 1 diabetes autoantibodies.. J Immunol Methods.

[24] Perfetto SP, Chattopadhyay PK, Roederer M (2004). Seventeen-colour flow cytometry: unravelling the immune system.. Nat Rev Immunol.

[25] Baecher-Allan C, Viglietta V, Hafler DA (2002). Inhibition of human CD4+CD25+high regulatory T cell function.. J Immunol.

[26] Glisic-Milosavljevic S, Waukau J, Jana S, Jailwala P, Rovensky J (2005). Comparison of apoptosis and mortality measurements in peripheral blood mononuclear cells PBMCs using multiple methods.. Cell Prolif.

[27] Hori S, Sakaguchi S (2004). Foxp3: a critical regulator of the development and function of regulatory T cells.. Microbes Infect.

[28] Federici M, Hribal M, Perego L, Ranalli M, Caradonna Z (2001). High glucose causes apoptosis in cultured human pancreatic islets of Langerhans: a potential role for regulation of specific Bcl family genes toward an apoptotic cell death program.. Diabetes.

[29] Otton R, Soriano FG, Verlengia R, Curi R (2004). Diabetes induces apoptosis in lymphocytes.. J Endocrinol.

[30] Baecher-Allan C, Wolf E, Hafler DA (2005). Functional analysis of highly defined, FACS-isolated populations of human regulatory CD4+ CD25+ T cells.. Clin Immunol.

[31] Tadokoro CE, Shakhar G, Shen S, Ding Y, Lino AC (2006). Regulatory T cells inhibit stable contacts between CD4+ T cells and dendritic cells in vivo.. J Exp Med.

[32] Thornton AM, Donovan EE, Piccirillo CA, Shevach EM (2004). Cutting edge: IL-2 is critically required for the in vitro activation of CD4+CD25+ T cell suppressor function.. J Immunol.

[33] Bennett CL, Christie J, Ramsdell F, Brunkow ME, Ferguson PJ (2001). The immune dysregulation, polyendocrinopathy, enteropathy, X-linked syndrome IPEX is caused by mutations of FOXP3.. Nat Genet.

[34] Fahlen L, Read S, Gorelik L, Hurst SD, Coffman RL (2005). T cells that cannot respond to TGF-beta escape control by CD4+CD25+ regulatory T cells.. J Exp Med.

[35] Fritzsching B, Oberle N, Eberhardt N, Quick S, Haas J (2005). In contrast to effector T cells, CD4+CD25+FoxP3+ regulatory T cells are highly susceptible to CD95 ligand- but not to TCR-mediated cell death.. J Immunol.

